# The potential of evaluating shape drawing using machine learning for predicting high autistic traits

**DOI:** 10.1371/journal.pone.0320770

**Published:** 2025-04-09

**Authors:** Yoshimasa Ohmoto, Kazunori Terada, Hitomi Shimizu, Akira Imamura, Ryoichiro Iwanaga, Hirokazu Kumazaki

**Affiliations:** 1 Faculty of Informatics, Department of Behavior Informatics, Shizuoka University, Shizuoka, Japan; 2 Faculty of Engineering, Department of Electrical, Electronic, and Computer Engineering, Gifu University, Gifu, Japan; 3 Department of Neuropsychiatry, Graduate School of Biomedical Sciences, Nagasaki University, Nagasaki, Japan; 4 Unit of Medical Science, Nagasaki University Graduate School of Biomedical Sciences, Nagasaki, Japan; Chiba Daigaku, JAPAN

## Abstract

**Background:**

Children with high autistic traits often exhibit deficits in drawing, an important skill for social adaptability. Machine learning is a powerful technique for learning predictive models from movement data, so drawing processes and product characteristics can be objectively evaluated. This study aimed to assess the potential of evaluating shape drawing using machine learning to predict high autistic traits.

**Method:**

Seventy boys (5.03 ± 0.16) and 63 girls (5.06 ± 0.18) from the general population participated in the study. Participants were asked to draw shapes in the following order: equilateral triangle, inverted equilateral triangle, square, and the sun. A model for classifying participants as likely to have high autistic traits was developed using a support vector machine algorithm with a linear kernel utilizing 16 variables. A 16-inch liquid crystal display pen tablet was used to acquire data on hand-finger fine motor activity while the participants drew each shape. The X and Y coordinates of the pen tip, pen pressure, pen orientation, pen tilt, and eye movements were recorded to determine whether the participants had any problems with this skill. Eye movements were assessed using a webcam. These data and eye movements were used to identify the variables for the support vector machine model.

**Data and Results:**

For each shape, a model support vector machine was created to classify the high and low autistic trait groups, with accuracy, sensitivity, and specificity all above 85%. The specificity values across all models were 100%. In the inverted equilateral triangle model, specificity, accuracy, and sensitivity values were 100%.

**Conclusions:**

These results demonstrate the potential of assessing shape characteristics using machine learning to predict high levels of autistic traits. Future studies with a wider variety of shapes are warranted to establish further the potential efficacy of drawing skills for screening for autism spectrum conditions.

## Introduction

Autism spectrum condition (ASC) is a developmental condition that affects social communication, social interactions, and repetitive restricted behavior. The signs and symptoms of ASC usually manifest during infancy or early childhood. Children with ASC may struggle with social interaction, communication, and repetitive behaviors and require extra support in educational settings. ASC is associated with a lifetime social cost of approximately $3.6 million [1].

ASC is considered a “spectrum condition” because of the wide variations in the type and severity of symptoms that children with ASC experience. Children with ASC exhibit various behavioral anomalies, each of which can be considered an independent dimension of ASC. Thus, ASC can be conceptualized as a multidimensional disorder, each dimension referring to a specific symptom (anomalous behavioral trait) of a given severity. The conceptualization of ASC as a heterogeneous multidimensional disorder has been addressed in previous studies [2–5]. Previous studies have revealed that a greater degree of autistic traits predicts emotional symptoms, peer problems [6], and more significant psychiatric difficulties [7]. The results of these studies highlight that in addition to clinical ASC, it is also important to focus on subthreshold autistic traits because autistic traits exist in a continuum and may be found in general non-clinical populations.

Increasing evidence suggests that early intervention programs, especially those implemented before elementary school, can improve social functioning in children with high autistic traits [8–11]. Although children with high autistic traits can be detected as early as 14 months [12] and with high certitude before two years of age [13], the latest prevalence reports reveal that more than 70% of affected children are not diagnosed before the age of 51 months [14]. Although the severity of autistic traits in many children is stable during early childhood, some children improve or worsen their autistic traits over time [2,5]. Previous studies revealed that autistic traits in five-year-olds predicted later emotional symptoms and peer problems [6]. It is important to identify autistic traits in all children who may undergo early screening before entering elementary school.

Generally, an assessment is time consuming. Most screening tests are questionnaire-based with low to moderate accuracy [15]. Furthermore, they are prone to recall and subjectivity biases [16]. Tools that can deliver objective and scalable quantification of behavioral atypicality are required to overcome these limitations.

Children with high autistic traits are commonly linked with early developmental delays in fine motor skills [17,18], often manifest as writing and drawing deficits [19,20]. As these skills are vital for social adaptability [21,22], it is important to emphasize drawing when screening children with high autistic traits. Kushki et al. [20] noted atypicalities in handwriting legibility and letter formation. Zajic and Wilson [23] reported that children with high autistic traits show challenges in literacy skills such as writing. Finnegan and Accardo [24] reported a lower spelling, handwriting, and composition performance. These studies support the idea that handwritten products of children with high autistic traits are less legible than those of children with low autistic traits [22,24]. Strategies for drawing equilateral triangles have been found to predict children’s handwriting abilities [25]. Thus, drawing an equilateral triangle could be a quick and easy technique for predicting high levels of autistic traits.

Given that fine motor skills change with age [17], drawing skills may also change. In our unpublished study, the task of drawing a triangle was too difficult to complete for many children younger than four. This confounding factor should be minimized by using subjects within a narrow age range restricted to five-year-olds. Previous studies [26] reported triangle drawing as an age-appropriate task, and the pass rate for five-year-olds was 51.2%. Based on previous studies [26] and our experience, differences in the progress of drawing skills become evident at age five, and as writing opportunities increase as children enter elementary school, this may be the appropriate age to use drawing to predict high autistic traits. Furthermore, considering the difficulties in executive function [27–29] and specificity in visual working memory [30] in children with high autistic traits, we proposed assessing the drawing of inverted equilateral triangles, squares, and the sun.

In addition to fine motor activities, inappropriate eye movements, such as unusual gaze patterns, stereotyped eye movements, avoidance of eye contact, and difficulty in joint attention, have been suggested to predict high autistic traits [23,31–33]. Given that writing pressure [34,35], speed [20,22,24,31,34], tilt [35], and orientation [36] are related to drawing features, it has been suggested that these elements are also related to shape-drawing skills in children with high autistic traits. However, the features of drawing shapes associated with autistic traits remain unclear. In our preliminary study (unpublished data), it was difficult to objectively evaluate the cause of awkwardness by observing the process of drawing a triangle.

Advanced computational approaches offer potential solutions to address the limitations of traditional assessment methods. Machine learning technologies have rapidly developed in recent years. Among these, neural network technology has significantly impacted various fields, such as movie recognition. Substantial research has been conducted on machine learning and children with high autistic traits [37–39]. Given that machine learning can be a powerful technique for predictive model learning from movement data, it is possible to objectively evaluate the drawing process and product characteristics using movies and machine learning to predict autistic traits.

In this study, we used a novel approach to predict high autistic traits by evaluating the task of drawing an equilateral triangle, an inverted equilateral triangle, a square, and the sun using machine learning. We conducted an experiment in a local preschool during the pupils’ medical examinations, with almost all five-year-old residents of Saza-cho (approximately 99%) participating. Conducting research in such a setting and targeting a narrow age range provides practical data. The Social Responsiveness Scale (SRS) is quickly accomplished, objective, economical, and easy to use and is increasingly used as a clinical screening tool for children with high autistic traits [40,41]. In the American Academy of Pediatrics guidelines for ASC, within the Diagnostic Evaluation section, the SRS is mentioned as a questionnaire to assess the severity of autistic traits [42]. In the present study, we used the standardized SRS score as the ground truth to determine the severity of autistic traits. This study aimed to assess the potential of using machine learning to evaluate drawings of an equilateral triangle, an inverted equilateral triangle, a square, and the sun to predict high autistic traits.

## Materials and methods

### Participants

Seventy boys (5.03 ± 0.16) and 63 girls (5.06 ± 0.18) from the general population participated in the study. This study was approved by the Ethics Committee of Nagasaki University, Japan (22051124). All the participants were recruited from Saza-cho, Nagasaki Prefecture, Japan. All procedures involving human participants were conducted by the institutional and/or national research committee’s ethical standards, the 1964 Declaration of Helsinki, and its subsequent amendments or comparable ethical standards. After receiving a complete explanation of the study, all the guardians provided written informed consent for participation. The inclusion criteria for the participants were: (1) aged five and (2) residents of Saza-cho. Participants were recruited between August 9, 2022, and August 6, 2024. The exclusion criterion was the inability to successfully acquire complete datasets (i.e., participants could not follow the instructions).

**Fig 1:**
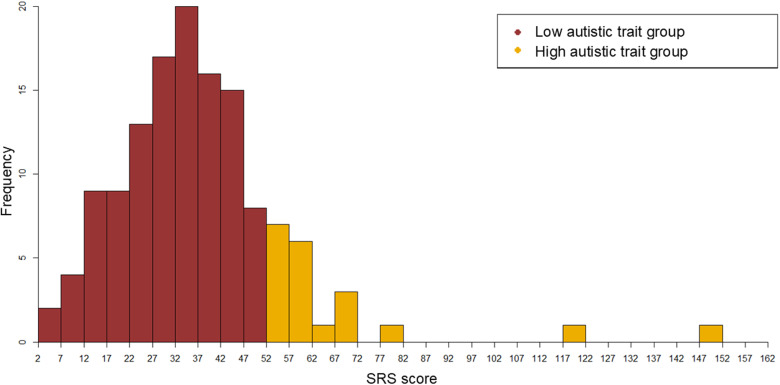
The distribution of the SRS scores in high autistic group and low autistic group.

The participants’ parents completed the SRS, Second Edition (SRS-2) [41] to predict high autistic traits and screen for clinically significant autistic symptoms. A higher SRS score indicated a higher degree of autistic traits (i.e., the high autistic trait group). We classified participants into high and low autistic trait groups based on the screening cutoff values (boys: 53.5, girls: 52.5) described in previous studies on the distribution of SRS scores in the Japanese population [43,44]. The high autistic trait group included 20 participants, and the low autistic trait group included 113 participants. The characteristics of the samples are listed in Table 1. Fig 1 depicts the distribution of the SRS scores in the two groups.

**Table 1:** Participant characteristics.

	High autistic trait group (n = 20) M (SD)	Low autistic trait group (n = 113) M (SD)	Statistics		
			t or χ^2^	df	*p*
Age	5.09 (0.17)	5.04 (0.17)	1.273	26	0.21
Male/female ratio	12: 8	58: 55	0.474	1	0.22
SRS-2	68.15 (23.68)	32.06 (11.26)	6.682	21	<0.001**

M., mean; SD, standard deviation; SRS-2, Social Responsiveness Scale, Second Edition.

** *p* <  0.001.

### Apparatus

A 16-inch liquid crystal display (LCD) pen tablet (Wacom Cintiq 16) was used to acquire hand–finger fine motor activity data while the participants drew each shape. We developed software using Unity3D (https://unity.com/) to provide the participants with exercises to familiarize them with the LCD pen tablet, which presented a video showing its shape and stroke order and measured the participants’ drawings. To estimate the eye movements for gaze tracking, facial images were captured using a webcam (Logicool c615n, 640 ×  480 pixels, 30 fps) placed on top of the LCD pen tablet. Data from the LCD pen tablet and the facial images were synchronously acquired at a recording rate of 30 Hz. A custom data acquisition program was developed to synchronize and record facial images from a webcam and draw data from the pen tablet.

### Procedure

**Fig 2:**
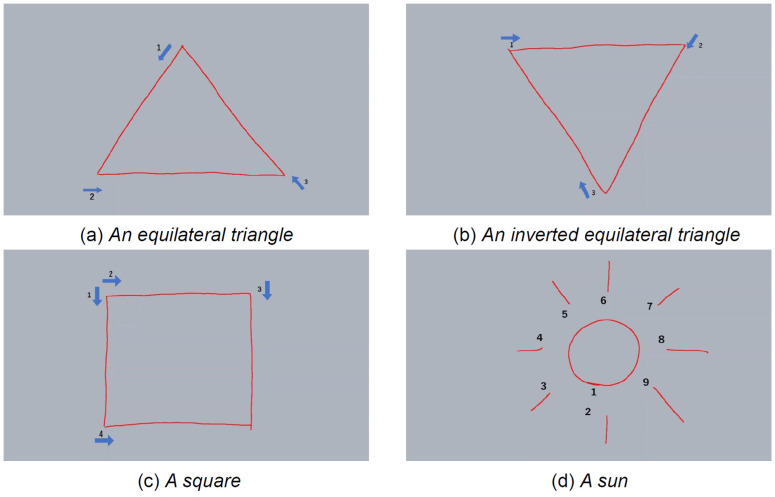
Drawing samples of (a) an equilateral triangle, (b) an inverted equilateral triangle, (c) a square, and (d) the sun in the LCD pen tablet. The numbers for each shape indicate the stroke order for each line to be drawn. These shapes were presented to the participants, and the drawing process was done via video during the data collection. LCD, liquid crystal display.

The experimenter sat with each participant. The pen was positioned horizontally in front of the LCD pen tablet so the participants could take it with their hand, which they habitually use to draw shapes in their daily activities. The experimenter presented a tablet screen and instructed the participants to draw on it. After approximately one minute of free drawing, the experimenter showed a demonstration video on the tablet screen and explained the shapes that would be drawn. The shapes were presented in a specific order to increase the complexity of the drawing gradually. The order was as follows: equilateral triangle, inverted equilateral triangle, square, and the sun (Fig 2). Each trial was performed once. The drawing video was created by asking the demonstrator to draw at a speed that was easy for a five-year-old child to imitate. The demonstration time was approximately 16 seconds for the equilateral triangle, 14 seconds for the inverted equilateral triangle, 15 seconds for the square, and 23 seconds for the sun. If the participant did not draw the presented shape or deviated significantly, the researcher made a judgment and eliminated the drawing information, prompting the participant to redraw the shape. When each shape was drawn, the experimenter presented a demonstration video explaining how to draw the next shape and prompted the participants to draw it.

### Measurements and collected data

**Fig 3:**
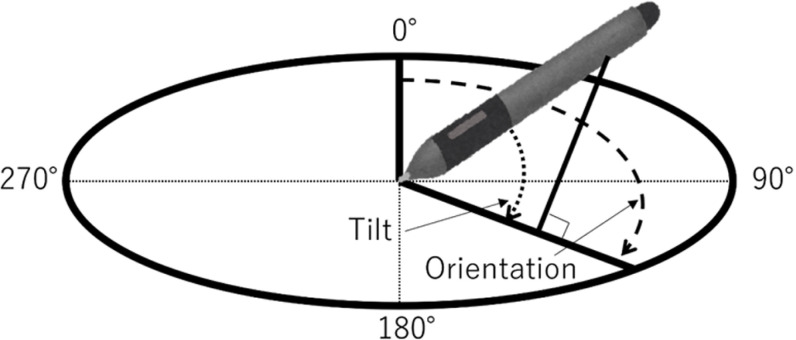
Tilt and orientation angles captured by the Wacom Cintiq 16.

The data collected in the experiment were time-series data of the X- and Y-coordinates of the pen tip, pen pressure, pen orientation, pen tilt, and eye movement (eye rotations around the X- and Y-axes) while drawing each shape. The pen orientation and tilt acquired from the pen tablet are shown in Fig 3. The X- and Y-coordinates of the pen tip, pen pressure, pen orientation, and pen tilt were measured by sensors in the Wacom Cintiq 16 system. The X and Y-coordinates of the pen tip were expressed as relative position coordinates concerning the tablet screen, taking values between 0 and 1. The pen pressure was taken at values between 0 and 1. The pen orientation was taken with values between 0 and 360 degrees. The pen tilt was taken with values between 0 and 90 degrees. Face images of participants were obtained from a camera mounted directly above the pen tablet screen. Eye rotation was estimated by processing the facial images using OpenFace [45]. The horizontal and vertical movements were taken at values between -90 and 90 degrees. An eye tracker could not be used effectively due to the close distance of approximately 40 cm during the shape-drawing tasks. Instead, a webcam was used because 40 cm was the appropriate distance for functionality.

### Classification by support vector machine

Sixteen features were extracted from the measured data and are listed in Table 2. As preprocessing, we extracted data segments corresponding to participants’ actively drawing shapes based on the trajectory data obtained from each shape drawing. See Supplementary Material 1 (S1 File) and Supplementary Material Table 1 (Table S1) for the reasons for choosing the variables and calculation formulas for each parameter after preprocessing.

**Table 2:** Variables for SVM.

Category	Variables	Explanation
Pen Pressure	$M_{penpressure}$	Mean of pen pressure
	$SD_{penpressure}$	The standard deviation of pen pressure
	$M_{penpressurechange}$	Mean change in pen pressure
Pen Tip Movement	_Drawingspeed_	Mean length of a line drawn per unit of time
	$SD_{drawingspeed}$	The standard deviation of drawing speed
	$M_{drawingacceleration}$	Mean change in drawing speed
Pen Barrel Pose	$M_{pentilt}$	Mean of pen tilt angle
	$SD_{pentilt}$	The standard deviation of pen tilt angle
	$M_{pentiltchange}$	Mean change in pen tilt angle
	$M_{penorientation}$	Mean of pen orientation angle
	$SD_{penorientation}$	The standard deviation of pen orientation angle
	$M_{penorientationchange}$	Mean change in pen orientation angle
Eye Movement Tracking in Demonstration	$Corr_{demoeyehorizontal}$	Correlation between eye movement tracking and advancing line movements in the horizontal direction while watching a demonstration of shape drawing
	$Corr_{demoeyevertical}$	Correlation between eye movement tracking and advancing line movements in the vertical direction while watching a demonstration of shape drawing
Eye Movement Tracking in Drawing Shape	$Corr_{drawingeyehorizontal}$	Correlation between eye movement tracking and self-drawing pen tip movements in the horizontal direction while drawing the shape
	$Corr_{drawingeyevertical}$	Correlation between eye movement tracking and self-drawing pen tip movements in the vertical direction while drawing the shape

SVM, support vector machine; unittime, the duration between drawing points $P_{t}$ and point $P_{t+1}$; drawing speed, length of the line drawn per unit time.

The support vector machine (SVM) is a popular machine-learning method in many classification problems. A SVM can be used to classify numerous objects [46]. We used 16 variables to perform classification learning using SVM to determine whether the participants belonged to the high or low autistic trait group. We selected variables from the 16 candidates described above and simultaneously optimized the hyperparameters for screening the high autistic trait group to improve the accuracy and generalization ability of the model. Classification models were built for each shape based on the drawing data. The probabilities of the high autistic trait group were calculated using an SVM model. The performances of the classification models were evaluated using leave-one-out cross-validation. For further information on the construction and evaluation of these models, see Supplementary Material 2 (S2 File).

### Statistical analysis

Statistical analyses were performed using the SPSS software (version 27.0; IBM, Armonk, NY, USA). Descriptive statistics were calculated for each sample. Differences in age and SRS-2 scores between the groups (i.e., the high and low autistic trait groups) were analyzed using an independent sample t-test. The difference in the sex ratio was analyzed using the χ^2^ test. We calculated the accuracy, sensitivity, and specificity based on the classification results of the leave-one-out cross-validation using the best classification model for each shape. The Shapley additive explanation (SHAP) value was used to identify important features of each shape. This computes the impact of each feature on the classifier’s output and the range of values of each feature to increase the probability of participants being classified into high or low-autistic trait groups. A significance threshold of *p* < .05 was adopted for all statistical tests.

## Results

Table 3 presents the classification results of the best classification models for each shape. The accuracy, sensitivity, and specificity were greater than 85% for each shape model. The specificity values across all models were 100%. In the inverted equilateral triangle model, specificity values, accuracy, and sensitivity were also 100%. When variable selection was performed from the features of all shapes, two features were selected from the models for individual shapes, and four features that were not included in the individual models were selected.

**Table 3:** The best leave-one-out cross-validation results.

	Accuracy	Sensitivity	Specificity	Selected Variables
**Equilateral triangle** (0.001)	0.985	0.900	1.000	High autistic trait group ~ $M_{penpressure}$ + $M_{penpressurechange}$ + $M_{drawingspeed}$ +
**Inverted equilateral triangle** (0.001)	1.000	1.000	1.000	High autistic trait group ~ $M_{penpressurechange}$ + $SD_{penpressure}$ + $M_{drawingacceleration}$ + $SD_{pentilt}$ + $M_{penorientationchange}$
**Square** (0.001)	0.985	0.900	1.000	High autistic trait group ~ $M_{penpressurechange}$ + $SD_{penpressure}$ + $M_{pentiltchange}$ + $SD_{pentilt}$ + $M_{penorientationchange}$ + $Corr_{demoeyehorizontal}$ + $Corr_{demoeyevartical}$ + $Corr_{drawingeyevartical}$
**The sun** (0.001)	0.977	0.850	1.000	High autistic trait group ~ $M_{penpressure}$ + $SD_{penpressure}$ + $M_{drawingspeed}$ + $M_{pentilt}$ + $M_{penorientation}$ + $Corr_{demoeyehorizontal}$ + $Corr_{drawingeyehorizontal}$ + $Corr_{drawingeyevartical}$
**All features** (0.001)	1.000	1.000	1.000	High autistic trait group ~ $Corr_{demoeyevartical}^{Equilateraltriangle}$ + $M_{penpressurechange}^{Invertedtriangle}$ + $SD_{drawingspeed}^{Invertedtriangle}$ + $M_{drawingacceleration}^{Invertedtriangle}$ + $M_{pentilt}^{Square}$ + $SD_{penorientation}^{Sun}$

LOOCV, leave-one-out cross-validation; SVM, support vector machine.

The numbers below the shape names represent the hyperparameters of the SVM model with a linear kernel. The value of is a *Cost* parameter. See Table 2 for the variable names.

The superscripts in ‘All features’ indicate that variables from each shape were selected.

The numbers below the shape names represent the hyperparameters of the SVM model with a linear kernel. The value of is a *Cost* parameter. See Table 2 for the variable names.

**Fig 4:**
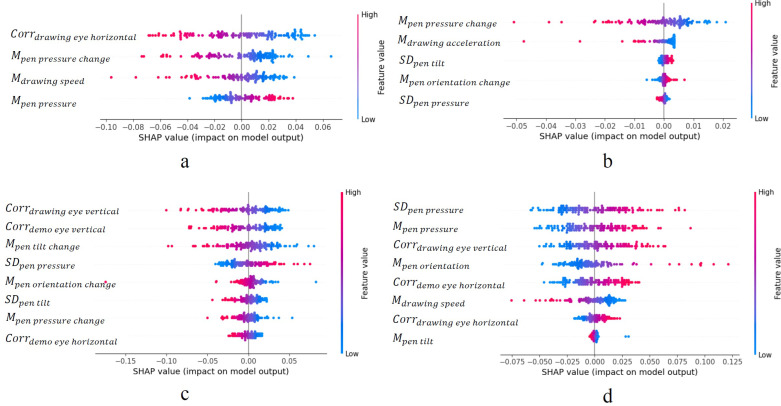
(a) The impact of each feature on the equilateral triangle model analyzed by SHAP. (b) The impact of each feature on the inverted equilateral triangle model analyzed by SHAP. (c) The impact of each feature on the square model analyzed by SHAP. (d) The impact of each feature on the sun model analyzed by SHAP.

The SHAP values of the selected features in each SVM model with impact are shown in Fig 4 for all participants and shapes. Each SVM model used four to eight variables. Even when the same variable was selected from different figures, its contribution to the classification varied among shapes. Except for the classification models of the inverted equilateral triangles, the variables related to eye movement were important (Fig 4a, c, and d). The pen pressure changes and drawing acceleration were important for the classification models of the inverted equilateral triangles (Fig 4b). For the sun classification models, the contributions of the employed variables were small and dispersed (Fig 4).

## Discussion

In this study, we assessed the potential of evaluating drawings of an equilateral triangle, an inverted equilateral triangle, a square, and the sun using machine learning to predict high autistic traits. We demonstrated that assessing the drawing characteristics of these shapes can achieve high accuracy, sensitivity, and specificity in identifying high autistic trait groups. The specificity values across all models were 100%. In the inverted equilateral triangle model, specificity, accuracy, and sensitivity values were also 100%. In the inverted equilateral triangle model, specificity, accuracy, and sensitivity values were 100%. Importantly, the screening took a very short time to complete. These results demonstrate the potential of shape drawing as a technique for ASC screening.

Our findings corroborate several aspects of previous research on handwriting analysis and autistic traits. Consistent with earlier studies [34,35], we observed the significance of writing pressure in all drawing tasks. Speed-related handwriting difficulties, reported in multiple studies [20,22,24,31], were also evident in our results for the equilateral triangle, inverted equilateral triangle, and sun drawings. Furthermore, our analysis of the inverted equilateral triangle, square, and sun drawings emphasized the importance of tilt in handwriting performance, consistent with the findings of Rosenblum et al. [33]. Finally, the relevance of handwriting orientation, as noted by Shin et al. [36], was supported by the features identified in our drawing models of the inverted equilateral triangle, square, and sun. These consistencies with prior research reinforce the validity of our approach and contribute to the growing body of evidence that links handwriting characteristics to autistic traits. Although there was some overlap in the selected variables between each shape, the impact of the variables analyzed using the SHAP values suggested that different features were selected from different shapes. The variables selected for predicting drawing characteristics differed for each shape, suggesting the importance of assessing the equilateral and inverted equilateral triangles, square, and sun. The fact that the variables selected by the individual shape model and the all-feature model did not match suggests this study captures rich information about the characteristics of ASC from various perspectives.

Our results on the variables related to eye movement are consistent with those of previous studies, suggesting the importance of eye movement in screening children with high autistic traits [23,31–33]. However, our method captured more detailed eye movement characteristics to identify patients with high autistic traits. For the equilateral triangle, eye movements were the key to classification while drawing the shape. In contrast, eye movements while observing the demonstration and drawing the shape were more important for the square and sun. The differences in the selected variables among the shapes may be attributed to the increased cognitive demands of the square and sun tasks, particularly executive function and visual working memory. These demands may accentuate the distinctions between high- and low-autistic trait groups. Executive function deficits in children with high autistic traits can lead to drawing and motor planning challenges, which may affect eye movement coordination and hand movements in some children.

Eye tracking is one of the most frequently used areas of machine learning for screening children with high autistic traits. According to a review article by Wei et al. [39], machine learning classification using eye tracking has an accuracy of 81%, specificity of 79%, and sensitivity of 84% in distinguishing individuals with ASC from TD individuals. These data support the idea that using drawing characteristics as features to screen for children with high autistic traits is realistic. In addition, the results of this study are relevant in practice, considering that the participants were limited to five-year-old children and it took a short time to conduct the drawing task. Furthermore, using tablets and movies makes it possible to perform the task at home, which is also promising for the future implementation of screening for children with high autistic traits.

The SRS was rated based on a subjective evaluation by the parents. However, the assessment in this study (i.e., shape assessment) was based on objective evaluation. Considering that the methods for assessing the SRS and shape are completely different, it is possible that assessing both is potentially more useful for screening children with high autistic traits.

This study had some limitations that should be addressed in future research. First, the sample size was relatively small. Second, the SRS-2 was used solely to classify the screening for the high and low autistic trait groups. Third, only four shapes were used in the drawing task. To address these limitations and advance understanding, we propose several directions for future research. First, studies with larger sample sizes are required to provide more robust and generalizable data on the potential use of drawing skills in screening children with high autistic traits. Second, incorporating more comprehensive measures of autistic traits, such as the Childhood Autism Rating Scale, is recommended to capture a broader range of autistic characteristics. Third, future studies should include various shapes and drawing tasks to identify more efficient and sensitive indicators for screening children with high autistic traits. Fourth, it may be possible that the dataset imbalance influences the performance of SVM. In future studies, increasing the number of participants can minimize the impact on SVM performance. Fifth, the typical machine learning method separates data to training validation and test sets. However, due to the nature and size of our dataset—and to make the most out of all available data—we choose LOOCV. Future studies to look at training, validation, and the out-of-the-sample performance of the model are needed. Sixth, the age range is highly restricted.

On the other hand, to minimize the impact of age as a confounding factor, we established the inclusion criteria for the participants restricted to five-year-olds. In addition, longitudinal studies are valuable for examining how drawing skills develop over time in individuals with high levels of autistic traits. Finally, investigating the relationship between drawing skills and other cognitive and motor abilities in children with high autistic traits may provide a more comprehensive understanding of the underlying mechanisms.

## Conclusions

This is the first study to assess the potential of evaluating drawing shapes (triangles, inverted equilateral triangles, squares, and the sun) using machine learning to screen children with high autistic traits. Our results demonstrate the usefulness of shape drawing as a simple and important approach for screening young children with high autistic traits. Future studies with a wider variety of shapes are warranted to establish further the potential efficacy of drawing skills in screening ASC.

## Supporting information

S1 Table Formulae for variables for SVM. (DOCX)

S1 File The reasons for choosing the variables and calculation methods. (DOCX)

S2 File Information on the construction and evaluation of classification models. (DOCX)

S3 File Data sheet Equirateral Triangle. (CSV)

S4 File Data sheet Invert Equirateral Triangle. (CSV)

S5 File Data sheet Sun. (CSV)

S6 File Data sheet Square. (CSV)

## References

[1] Cakir J, Frye RE, Walker SJ. The lifetime social cost of autism: 1990–2029. Research in Autism Spectrum Disorders. 2020;72:101502. doi: 10.1016/j.rasd.2019.101502

[2] Gotham K, Pickles A, Lord C. Trajectories of autism severity in children using standardized ADOS scores. Pediatrics. 2012;130(5):e1278-84. doi: 10.1542/peds.2011-3668 23090336 PMC3483889

[3] Matson JL, Goldin RL. What is the future of assessment for autism spectrum disorders: Short and long term. Research in Autism Spectrum Disorders. 2014;8(3):209–13. doi: 10.1016/j.rasd.2013.01.007

[4] Mehling MH, Tassé MJ. Severity of Autism Spectrum Disorders: Current Conceptualization, and Transition to DSM-5. J Autism Dev Disord. 2016;46(6):2000–16. doi: 10.1007/s10803-016-2731-7 26873143

[5] Venker CE, Ray-Subramanian CE, Bolt DM, Ellis Weismer S. Trajectories of autism severity in early childhood. J Autism Dev Disord. 2014;44(3):546–63. doi: 10.1007/s10803-013-1903-y 23907710 PMC3909724

[6] Saito A, Stickley A, Haraguchi H, Takahashi H, Ishitobi M, Kamio Y. Association Between Autistic Traits in Preschool Children and Later Emotional/Behavioral Outcomes. J Autism Dev Disord. 2017;47(11):3333–46. doi: 10.1007/s10803-017-3245-7 28785972 PMC5633642

[7] Kanne SM, Christ SE, Reiersen AM. Psychiatric symptoms and psychosocial difficulties in young adults with autistic traits. J Autism Dev Disord. 2009;39(6):827–33. doi: 10.1007/s10803-008-0688-x 19132522

[8] Reichow B. Overview of meta-analyses on early intensive behavioral intervention for young children with autism spectrum disorders. J Autism Dev Disord. 2012;42(4):512–20. doi: 10.1007/s10803-011-1218-9 21404083

[9] Rogers SJ, Estes A, Lord C, Munson J, Rocha M, Winter J, et al. A Multisite Randomized Controlled Two-Phase Trial of the Early Start Denver Model Compared to Treatment as Usual. J Am Acad Child Adolesc Psychiatry. 2019;58(9):853–65. doi: 10.1016/j.jaac.2019.01.004 30768394 PMC12088912

[10] Smith T, Iadarola S. Evidence Base Update for Autism Spectrum Disorder. J Clin Child Adolesc Psychol. 2015;44(6):897–922. doi: 10.1080/15374416.2015.1077448 26430947

[11] Warren Z, McPheeters ML, Sathe N, Foss-Feig JH, Glasser A, Veenstra-Vanderweele J. A systematic review of early intensive intervention for autism spectrum disorders. Pediatrics. 2011;127(5):e1303-11. doi: 10.1542/peds.2011-0426 21464190

[12] Landa RJ, Gross AL, Stuart EA, Faherty A. Developmental trajectories in children with and without autism spectrum disorders: The first 3 years. Child Dev. 2013;84: 429-442. doi: 10.1111/j.1467-8624.2012.01870.x PMC4105265 23110514

[13] Ozonoff S, Young GS, Landa RJ, Brian J, Bryson S, Charman T, et al. Diagnostic stability in young children at risk for autism spectrum disorder: a baby siblings research consortium study. J Child Psychol Psychiatry. 2015;56(9):988–98. doi: 10.1111/jcpp.12421 25921776 PMC4532646

[14] Maenner MJ, Warren Z, Williams AR, Amoakohene E, Bakian AV, Bilder DA, et al. Prevalence and Characteristics of Autism Spectrum Disorder Among Children Aged 8 Years - Autism and Developmental Disabilities Monitoring Network, 11 Sites, United States, 2020. MMWR Surveill Summ. 2023;72(2):1–14. doi: 10.15585/mmwr.ss7202a1 36952288 PMC10042614

[15] Yuen T, Penner M, Carter MT, Szatmari P, Ungar WJ. Assessing the accuracy of the Modified Checklist for Autism in Toddlers: a systematic review and meta-analysis. Dev Med Child Neurol. 2018;60(11):1093–100. doi: 10.1111/dmcn.13964 29992541

[16] Barton ML, Dumont-Mathieu T, Fein D. Screening young children for autism spectrum disorders in primary practice. J Autism Dev Disord. 2012;42(6):1165–74. doi: 10.1007/s10803-011-1343-5 21842325

[17] Bhat A. Multidimensional motor performance in children with autism mostly remains stable with age and predicts social communication delay, language delay, functional delay, and repetitive behavior severity after accounting for intellectual disability or cognitive delay: A SPARK dataset analysis. Autism Res. 2023;16(1):208–29. doi: 10.1002/aur.2870 36533674 PMC9939031

[18] Moruzzi S, Ogliari A, Ronald A, Happé F, Battaglia M. The nature of covariation between autistic traits and clumsiness: a twin study in a general population sample. J Autism Dev Disord. 2011;41(12):1665–74. doi: 10.1007/s10803-011-1199-8 21347613

[19] Casellato C, Zorzi G, Pedrocchi A, Ferrigno G, Nardocci N. Reaching and writing movements: sensitive and reliable tools to measure genetic dystonia in children. J Child Neurol. 2011;26(7):822–9. doi: 10.1177/0883073810392997 21421904

[20] Kushki A, Chau T, Anagnostou E. Handwriting difficulties in children with autism spectrum disorders: a scoping review. J Autism Dev Disord. 2011;41(12):1706–16. doi: 10.1007/s10803-011-1206-0 21350917

[21] Chen Y, Fei X, Wu T, Li H, Xiong N, Shen R, et al. The relationship between motor development and social adaptability in autism spectrum disorder. Front Psychiatry. 2022;13:1044848. doi: 10.3389/fpsyt.2022.1044848 36506435 PMC9726915

[22] Grace N, Enticott PG, Johnson BP, Rinehart NJ. Do Handwriting Difficulties Correlate with Core Symptomology, Motor Proficiency and Attentional Behaviours?. J Autism Dev Disord. 2017;47(4):1006–17. doi: 10.1007/s10803-016-3019-7 28083779

[23] Zajic MC, Wilson SE. Writing research involving children with autism spectrum disorder without a co-occurring intellectual disability: A systematic review using a language domains and mediational systems framework. Research in Autism Spectrum Disorders. 2020;70:101471. doi: 10.1016/j.rasd.2019.101471

[24] Finnegan E, Accardo AL. Written Expression in Individuals with Autism Spectrum Disorder: A Meta-Analysis. J Autism Dev Disord. 2018;48(3):868–82. doi: 10.1007/s10803-017-3385-9 29164435

[25] Tabatabaey–Mashadi N, Sudirman R, Khalid PI. An Evaluation of Children’s Structural Drawing Strategies. Jurnal Teknologi. 2013;61(2). doi: 10.11113/jt.v61.1632

[26] Tanaka Educational Research Institute. editor. Tanaka-Binet intelligence test VI: Theoretical manual. Tanken Publishing.

[27] Lee RR, Ward AR, Lane DM, Aman MG, Loveland KA, Mansour R, et al. Executive Function in Autism: Association with ADHD and ASD Symptoms. J Autism Dev Disord. 2023;53(2):688–700. doi: 10.1007/s10803-020-04852-2 33515417 PMC8322145

[28] Ring M, Gaigg SB, de Condappa O, Wiener JM, Bowler DM. Spatial navigation from same and different directions: The role of executive functions, memory and attention in adults with autism spectrum disorder. Autism Res. 2018;11(5):798–810. doi: 10.1002/aur.1924 29405653

[29] Zhang Z, Peng P, Zhang D. Executive Function in High-Functioning Autism Spectrum Disorder: A Meta-analysis of fMRI Studies. J Autism Dev Disord. 2020;50(11):4022–38. doi: 10.1007/s10803-020-04461-z 32200468

[30] Funabiki Y, Shiwa T. Weakness of visual working memory in autism. Autism Res. 2018;11(9):1245–52. doi: 10.1002/aur.1981 30260579

[31] Hellinckx T, Roeyers H, Van Waelvelde H. Predictors of handwriting in children with Autism Spectrum Disorder. Research in Autism Spectrum Disorders. 2013;7(1):176–86. doi: 10.1016/j.rasd.2012.08.009

[32] Kaiser M-L, Albaret J-M, Doudin P-A. Relationship Between Visual-Motor Integration, Eye-Hand Coordination, and Quality of Handwriting. Journal of Occupational Therapy, Schools, & Early Intervention. 2009;2(2):87–95. doi: 10.1080/19411240903146228

[33] Rosenblum S, Simhon HAB, Gal E. Unique handwriting performance characteristics of children with high-functioning autism spectrum disorder. Research in Autism Spectrum Disorders. 2016;23 235–44. doi: 10.1016/j.rasd.2015.11.004

[34] Li-Tsang CWP, Li TMH, Ho CHY, Lau MSW, Leung HWH. The Relationship Between Sensorimotor and Handwriting Performance in Chinese Adolescents with Autism Spectrum Disorder. J Autism Dev Disord. 2018;48(9):3093–100. doi: 10.1007/s10803-018-3580-3 29675766

[35] Verma P, Khandelwal KB, Sharma P, Lahiri U. Investigating the role of graphic pressure and temporal measures in influencing graphic skills of individuals with autism using a digital platform. J Ambient Intell Human Comput. 2023;14(10):14249–59. doi: 10.1007/s12652-023-04663-0

[36] Shin J, Maniruzzaman Md, Uchida Y, Hasan MdAM, Megumi A, Yasumura A. Handwriting-Based ADHD Detection for Children Having ASD Using Machine Learning Approaches. IEEE Access. 2023;11:84974–84. doi: 10.1109/access.2023.3302903

[37] Minissi ME, Chicchi Giglioli IA, Mantovani F, Alcañiz Raya M. Assessment of the Autism Spectrum Disorder Based on Machine Learning and Social Visual Attention: A Systematic Review. J Autism Dev Disord. 2022;52(5):2187–202. doi: 10.1007/s10803-021-05106-5 34101081 PMC9021060

[38] Moreau C, Deruelle C, Auzias G. Machine learning for neurodevelopmental disorders. In: Colliot O, editor. Machine learning for brain disorders. Neuromethods. New York: Springer US; 2023. pp. 977-1007. doi: 10.1007/978-1-0716-3195-9_31 37988540

[39] Wei Q, Cao H, Shi Y, Xu X, Li T. Machine learning based on eye-tracking data to identify Autism Spectrum Disorder: A systematic review and meta-analysis. J Biomed Inform. 2023;137:104254. doi: 10.1016/j.jbi.2022.104254 36509416

[40] Cholemkery H, Kitzerow J, Rohrmann S, Freitag CM. Validity of the social responsiveness scale to differentiate between autism spectrum disorders and disruptive behaviour disorders. Eur Child Adolesc Psychiatry. 2014;23(2):81–93. doi: 10.1007/s00787-013-0427-5 23719758

[41] Constantino J, Gruber C. The social responsiveness scale. Los Angeles: Western Psychological Services; 2001.

[42] Hyman SL, Levy SE, Myers SM, Kuo DZ, Apkon S, Davidson LF. Identification, Evaluation, and Management of Children With Autism Spectrum Disorder. Pediatrics. 2020;145(1):e20193447. doi: 10.1542/peds.2019-3447 31843864

[43] Kamio Y, Inada N, Moriwaki A, Kuroda M, Koyama T, Tsujii H, et al. Quantitative autistic traits ascertained in a national survey of 22 529 Japanese schoolchildren. Acta Psychiatr Scand. 2013;128: 45-53. doi: 10.1111/acps.12034 PMC3604131 23171198

[44] Shirama A, Stickley A, Kamio Y, Nakai A, Takahashi H, Saito A, et al. Emotional and behavioral problems in Japanese preschool children with motor coordination difficulties: the role of autistic traits. Eur Child Adolesc Psychiatry. 2022;31(6):979–90. doi: 10.1007/s00787-021-01732-7 33566188

[45] Baltrusaitis T, Zadeh A, Lim YC, Morency L-P. OpenFace 2.0: Facial Behavior Analysis Toolkit. 2018 13th IEEE International Conference on Automatic Face & Gesture Recognition (FG 2018). 2018:59–66. doi: 10.1109/fg.2018.00019

[46] Eman D, Emanuel AWR. Machine Learning Classifiers for Autism Spectrum Disorder: A Review. 2019 4th International Conference on Information Technology, Information Systems and Electrical Engineering (ICITISEE). 2019:255–60. doi: 10.1109/icitisee48480.2019.9003807

