# Supplementary material for: The potential of evaluating shape drawing using machine learning for predicting high autistic traits
Source: PLoS One. 2025 Apr 9;20(4):e0320770. doi: 10.1371/journal.pone.0320770 (PMC11981181; doi:10.1371/journal.pone.0320770)
Supplement: S1 Table — (DOCX) [file pone.0320770.s001.docx]

**Table S1*.* Formulae for explanatory variables for SVM**

| Category | Variables | Formula |
| --- | --- | --- |
| Pen Pressure | $M_{pen pressure}$ | $\frac{1}{n}\sum_{i=1}^{n} {PP}_{i}$,  where ${PP}_{i} is a pen pressure value (0\leq{PP}_{i}\leq1$) |
|  | ${SD}_{pen pressure}$ | $\sqrt{\frac{1}{n}\sum_{i=1}^{n} {{(PP}_{i}-M_{pen pressure})}^{2}}$,  where ${PP}_{i} is a pen pressure value (0\leq{PP}_{i}\leq1)$ |
|  | $M_{pen pressure change}$ | $\frac{1}{n-1}\sum_{i=1}^{n-1} {CPP}_{i}$,  ${CPP}_{i}={PP}_{i+1}-{PP}_{i} \left( 1\leq i\leq n-1 \right)$,  where ${PP}_{i} is a pen pressure value (0\leq{PP}_{i}\leq1)$ |
| Pen Tip Movement | $M_{drawing speed}$ | $\frac{1}{n-1}\sum_{i=1}^{n-1} {DS}_{i}$,  ${DS}_{i}=\frac{\sqrt{\left( h_{i+1}-h_{i} \right)^{2}+\left( v_{i+1}-v_{i} \right)^{2}}}{unit time} \left( 1\leq i\leq n-1 \right)$,  where $h_{i} is a horizontal position (0\leq h_{i}\leq1)$, $v_{i} is a vertical position (0\leq v_{i}\leq1)$ |
|  | ${SD}_{drawing speed}$ | $\sqrt{\frac{1}{n-1}\sum_{i=1}^{n-1} \left( {DS}_{i}-M_{drawing speed} \right)^{2}}$ |
|  | $M_{drawing acceleration}$ | $\frac{1}{n-2}\sum_{i=1}^{n-2} {ACC}_{i}$,  ${ACC}_{i}={DS}_{i+1}-{DS}_{i} (1\leq i\leq n-2)$ |

| Category | Variables | Formula |
| --- | --- | --- |
| Pen Barrel Pose | $M_{pen tilt}$ | $\frac{1}{n}\sum_{i=1}^{n} {PT}_{i}$,  where ${PT}_{i} is a pen tilt angle (0^{\circ}\leq{PT}_{i}\leq90^{\circ})$ |
|  | ${SD}_{pen tilt}$ | $\sqrt{\frac{1}{n}\sum_{i=1}^{n} {{(PT}_{i}-M_{pen tilt})}^{2}}$,  where ${PT}_{i} is a pen tilt angle (0^{\circ}\leq{PT}_{i}\leq90^{\circ})$ |
|  | $M_{pen tilt change}$ | $\frac{1}{n-1}\sum_{i=1}^{n-1} {CPT}_{i}$,  ${{CPT}_{i}=PT}_{i+1}-{PT}_{i} (1\leq i\leq n-1)$,  where ${PT}_{i} is a pen tilt angle (0^{\circ}\leq{PT}_{i}\leq90^{\circ})$ |
|  | $M_{pen orientation}$ | $\frac{1}{n}\sum_{i=1}^{n} {PO}_{i}$,  where ${PO}_{i} is a pen orientation angle (0^{\circ}\leq{PO}_{i}\leq360^{\circ})$ |
|  | ${SD}_{pen orientation}$ | $\sqrt{\frac{1}{n}\sum_{i=1}^{n} {{(PO}_{i}-M_{pen orientation})}^{2}}$,  where ${PO}_{i} is a pen orientation angle (0^{\circ}\leq{PO}_{i}\leq360^{\circ})$ |
|  | $M_{pen orientation change}$ | $\frac{1}{n-1}\sum_{i=1}^{n-1} {CPO}_{i}$,  ${{CPO}_{i}=PO}_{i+1}-{PO}_{i} (1\leq i\leq n-1)$,  where ${PO}_{i} is a pen orientation angle (0^{\circ}\leq{PO}_{i}\leq360^{\circ})$ |

| Category | Variables | Formula |
| --- | --- | --- |
| Gaze Tracking in Demonstration | ${Corr}_{demo gaze horizontal}$ | $\frac{\frac{1}{n}\sum_{i=1}^{n} {(a}_{i}-\frac{\sum_{k=1}^{n} a_{k}}{n}){(b}_{i}-\frac{\sum_{k=1}^{n} b_{k}}{n})}{\sqrt{\frac{1}{n}\sum_{i=1}^{n} {{(a}_{i}-\frac{\sum_{k=1}^{n} a_{k}}{n})}^{2}}\sqrt{\frac{1}{n}\sum_{i=1}^{n} {{(b}_{i}-\frac{\sum_{k=1}^{n} b_{k}}{n})}^{2}}}$,  where $a_{i} and a_{k} are horizontal movements of gaze$  $\left( -90^{\circ}\leq a_{i}, a_{k}\leq90^{\circ} \right),$  $b_{i} , b_{k} are horizontal movements of$  $advancing line in demo$  $(-1\leq b_{i}, b_{k}\leq1)$ |
|  | ${Corr}_{demo gaze vertical}$ | $\frac{\frac{1}{n}\sum_{i=1}^{n} {(a}_{i}-\frac{\sum_{k=1}^{n} a_{k}}{n}){(b}_{i}-\frac{\sum_{k=1}^{n} b_{k}}{n})}{\sqrt{\frac{1}{n}\sum_{i=1}^{n} {{(a}_{i}-\frac{\sum_{k=1}^{n} a_{k}}{n})}^{2}}\sqrt{\frac{1}{n}\sum_{i=1}^{n} {{(b}_{i}-\frac{\sum_{k=1}^{n} b_{k}}{n})}^{2}}}$,  where $a_{i} and a_{k} are vertical movements of gaze$  $\left( -90^{\circ}\leq a_{i}, a_{k}\leq90^{\circ} \right),$  $b_{i} and b_{k} are vertical movements of$  $advancing line in demo$  $(-1\leq b_{i}, b_{k}\leq1\boldsymbol{)}$ |
| Gaze Tracking in Drawing Shape | ${Corr}_{drawing gaze horizontal}$ | $\frac{\frac{1}{n}\sum_{i=1}^{n} {(a}_{i}-\frac{\sum_{k=1}^{n} a_{k}}{n}){(b}_{i}-\frac{\sum_{k=1}^{n} b_{k}}{n})}{\sqrt{\frac{1}{n}\sum_{i=1}^{n} {{(a}_{i}-\frac{\sum_{k=1}^{n} a_{k}}{n})}^{2}}\sqrt{\frac{1}{n}\sum_{i=1}^{n} {{(b}_{i}-\frac{\sum_{k=1}^{n} b_{k}}{n})}^{2}}}$,  where $a_{i} and a_{k} are horizontal movements of gaze$  $\left( -90^{\circ}\leq a_{i}, a_{k}\leq90^{\circ} \right),$  $b_{i} and b_{k} are horizontal movements of$  $drawing pen tip (-1\leq b_{i}, b_{k}\leq1\boldsymbol{)}$ |
|  | ${Corr}_{drawing gaze vertical}$ | $\frac{\frac{1}{n}\sum_{i=1}^{n} {(a}_{i}-\frac{\sum_{k=1}^{n} a_{k}}{n}){(b}_{i}-\frac{\sum_{k=1}^{n} b_{k}}{n})}{\sqrt{\frac{1}{n}\sum_{i=1}^{n} {{(a}_{i}-\frac{\sum_{k=1}^{n} a_{k}}{n})}^{2}}\sqrt{\frac{1}{n}\sum_{i=1}^{n} {{(b}_{i}-\frac{\sum_{k=1}^{n} b_{k}}{n})}^{2}}}$,  where $a_{i} and a_{k} are vertical movements of gaze$  $\left( -90^{\circ}\leq a_{i}, a_{k}\leq90^{\circ} \right),$  $b_{i} and b_{k} are vertical movements of$  $drawing pen tip (-1\leq b_{i}, b_{k}\leq1\boldsymbol{)}$ |

SVM, support vector machine; *n,* total number of data points for each participant;

$unit time,=$the duration between the drawing points $P_{t}$ and point $P_{t+1}$.

Pen Pressure

Variables related to pen pressure are computed based on pressure data recorded during drawing. The mean and standard deviation of the pressure values are calculated to quantify pressure variation. Additionally, the rate of change in pen pressure over time is measured to analyze pressure fluctuations.

Pen Tip Movement

Variables related to pen tip movement are derived from the coordinates of consecutive drawing points. The movement speed is calculated using the distance between successive points and the time interval. Acceleration is computed as the rate of change in speed, allowing for the evaluation of pen motion characteristics. Furthermore, curvature is measured to quantify the shape of the drawn trajectory.

Pen Barrel Pose

Variables related to the pen barrel pose are calculated based on sensor data. The pen tilt angle is measured and analyzed for changes over time. The angular velocity of rotation is computed using sensor data to quantify rotational motion. Additionally, the standard deviation of the pen tilt angle is determined to assess variations in inclination.

Gaze Tracking

Variables related to gaze tracking are computed based on estimated eye movement data by Openface. The eye movements are recorded during both the demonstration and drawing phases. The speed of eye movement is calculated from changes in estimated eye movements over time. Additionally, the eye movements and the pen tip position are computed to quantify the movement relationship between eye and pen movement.
