# Supplementary material for: The potential of evaluating shape drawing using machine learning for predicting high autistic traits
Source: PLoS One. 2025 Apr 9;20(4):e0320770. doi: 10.1371/journal.pone.0320770 (PMC11981181; doi:10.1371/journal.pone.0320770)
Supplement: S1 File — (DOCX) [file pone.0320770.s002.docx]

Supplementary Material

# Supplementary Material 1: The reasons for choosing the variables and calculation methods

We chose the mean and standard deviation of pen pressure as indicators of overall distribution ($M_{pen pressure}$ and ${SD}_{pen pressure}$) and the mean change in pen pressure as an indicator of short-term variability ($M_{pen pressure change}$), referring to previous studies (Li-tang et al., 2018; Verma et al., 2023; Shin et al., 2023).

We chose the mean and standard deviation ($M_{drawing speed}$ and ${SD}_{drawing speed}$) of the drawing speed, which was calculated as the length of the line drawn per unit time by using the pen tip movements, as indicators of the overall distribution. We used the mean change ($M_{drawing acceleration}$), in drawing speed, that is, the drawing acceleration, as an indicator of short-term variability based on previous studies (Kushki, et al., 2011; Hellinckx et al., 2013; Finnegan and Accardo, 2017; Grace et al., 2017; Li-tang et al., 2018; Shin et al., 2023).

We chose the mean and standard deviation ($M_{pen tilt}$ and ${SD}_{pen tilt}$) of the pen tilt angle, which was the pen barrel position relative to the vertical axis, as indicators of overall distribution, and the mean change in pen tilt angle ($M_{pen tilt change}$) as an indicator of short-term variability, referring to previous studies (Rosenblum et al., 2016; Shin et al., 2023).

We chose the mean and the standard deviation ($M_{pen orientation}$ and ${SD}_{pen orientation}$) of pen orientation angle, which was the pen barrel position in the horizontal plane as indicators of overall distribution, and the mean change in pen orientation angle ($M_{pen orientation change}$) as an indicator of short-term variability, referring to a previous study (Shin et al., 2023).

We chose the correlations between gaze tracking and advancing line movements (${Corr}_{demo gaze horizontal}$ and ${Corr}_{demo gaze vertical}$) in the demonstration in both the horizontal and vertical directions while watching the demonstration of the shape drawing as indicators of attention, referring to a previous study (Li and Ouyang, 2018) that attempted to classify suspected ASD and TD based on children's eye movement while watching a video.

We chose the correlations between gaze tracking and self-drawing pen tip movements (${Corr}_{drawing gaze horizontal}$ and ${Corr}_{drawing gaze vertical}$) in both the horizontal and vertical directions while drawing the shape presented in the demonstration as indicators of visual–motor integration, referring to previous studies that used the VMI test (Beery, 2004) to assess visual–motor integration in ASD (Kaiser, et al., 2009; Hellinckx et al., 2013; Rosenblum et al., 2019; Zajic et al., 2020).

*Note.* The mean values were selected as parameters to detect deviations from standard values between children with high and low autistic traits. The standard deviations were selected as parameters based on our prediction that children with high autistic traits would show greater variability due to their difficulties with fine motor control.

See **Table S1** for the calculation formulae of each explanatory variable.
