# Supplementary material for: The potential of evaluating shape drawing using machine learning for predicting high autistic traits
Source: PLoS One. 2025 Apr 9;20(4):e0320770. doi: 10.1371/journal.pone.0320770 (PMC11981181; doi:10.1371/journal.pone.0320770)
Supplement: S2 File — (DOCX) [file pone.0320770.s003.docx]

**Supplementary Material 2:** **Information on the construction and evaluation of classification models**

- 1. **Classification model training to predict** **high autistic trait**

The model for classifying whether participants were having high autistic trait was trained using a support vector machine (SVM) algorithm with the 16 explanatory variables (Tables 2 and S1). We used the ksvm function from the Kernlab library in R version 4.2.3 to run the SVM, using the linear kernel. In the linear kernel, there is a hyperparameter (cost). A grid search was performed to determine optimal hyperparameters. The search range was [0.001, 0.005, 0.01, 0.05, 0.1, 0.5, 1, 5, 10] for cost. To reduce computational time, variable selection was performed using the backward-forward selection method for each hyperparameter. For variable selection, five-fold cross-validation errors were compared, and the variables with the smallest cross-validation errors were chosen. A search for the hyperparameter and variables was performed for each shape.

- 1. **Performance evaluation of classification models**

After the training, the performance of the final classification model was evaluated.

In the optimization of the hyperparameter by the grid search described above, 5-fold cross-validation was used for model evaluation to reduce computation time. To evaluate the performance of the hyperparameter and variable sets more accurately, leave-one-out cross-validation (LOOCV) was performed on the top 50 hyperparameter and variable sets with the lowest cross-validation errors.

During the LOOCV process, the data for each participant were sequentially removed from the dataset. The SVM classification model was trained using the remaining data. Subsequently, the performance of the model was tested using data that were initially removed.
